# PREDOMOS study, impact of a social intervention program for socially isolated elderly cancer patients: update to the study protocol for a randomized controlled trial

**DOI:** 10.1186/s13063-018-3127-0

**Published:** 2019-01-15

**Authors:** Anne-Laure Couderc, Emilie Nouguerède, Karine Baumstarck, Sandrine Loubière, Hervé Le Caer, Olivier Guillem, Frédérique Rousseau, Laurent Greillier, Emmanuelle Norguet-Monnereau, Maud Cecile, Rabia Boulahssass, Françoise Le Caer, Sandrine Tournier, Chantal Butaud, Pierre Guillet, Sophie Nahon, Sylvie Kirscher, Nadine Diaz, Claire Morando, Patrick Villani, Pascal Auquier, Aurélie Daumas

**Affiliations:** 1 Service de Médecine Interne Gériatrie et Thérapeutique, CHU Sainte Marguerite, Assistance Publique des Hôpitaux de Marseille (AP-HM), 270 Boulevard de Sainte Marguerite Dromel, 13274 Marseille, cedex 09 France; 2 Unit of Coordination in Onco-Geriatry (UCOG), PACA-west, France; 3 Service d’Oncologie Digestive, CHU Timone, AP-HM, 264 Rue Saint Pierre, 13385 Marseille, cedex 05 France; 4 EA3279, Self-perceived Health Assessment Research Unit, Aix-Marseille University, 27 Boulevard Jean Moulin, 13385 Marseille, cedex 05 France; 5 Service de Pneumologie, CH Yves le Foll, 10 rue Marcel Proust, Saint-Brieuc, France; 6 Service d’Onco-Gériatrie, CH Intercommunal des Alpes du Sud Site de Gap (CHICAS), 1 Place Auguste Muret, 05000 Gap, France; 7 Service d’Oncologie Médicale, Institut Paoli Calmettes, 232 Boulevard de Sainte Marguerite Dromel, 13009 Marseille, France; 8 Oncologie Multidisciplinaire et Innovation Thérapeutique, CHU NORD, AP-HM, Chemin des Bourrely, 13915 Marseille, cedex 20 France; 9 Service de Gérontologie, Hôpital de Cimiez, 4 Avenue Reine Victoria, CS 91179, 06003 Nice, France; 10 Service de Gériatrie, CH Yves le Foll, 10 rue Marcel Proust, Saint-Brieuc, France; 11 Service de Gériatrie, Hôpital Saint Joseph, 26 Boulevard Louvain, 13285 Marseille, cedex 08 France; 12 Unité Mobile de Gériatrie, Hôpital Saint Musse, CH Intercommunal Toulon-La Seyne sur Mer (CHITS), 54 Rue Henri Claire Deville, 83000 Toulon, France; 13 Service d’Hémato-Oncologie, CH du Pays d’Aix, Avenue les Tamaris, 13616 Aix-en-Provence, France; 14 Service d’Oncologie Médicale, Institut Sainte Catherine (ISC), 250 Chemin de Baigne Pieds, 84918 Avignon, cedex 09 France; 15 Service Social, Hôpital Sainte Marguerite, AP-HM, 270 Boulevard de Sainte Marguerite Dromel, 13274 Marseille, cedex 09 France; 16 Direction de la Recherche Clinique et de l’Innovation (DRCI), AP-HM, 80 Rue Brochier, 13354 Marseille, cedex 05 France

**Keywords:** Social intervention program, Techniques of domotic and remote assistance, Older patient, cancer, Oncogeriatrics, Randomized controlled trial, Cost effectiveness

## Abstract

**Background:**

Social isolation potentiates the risk of death by cancer in the older cancer patient population. The PREDOMOS study investigates the impact of establishing a Program of Social intervention associated with techniques of Domotic and Remote assistance on the improvement of quality of life of older isolated patients, treated for locally advanced or metastatic cancer. This paper updates the pilot trial protocol.

**Methods/design:**

The original protocol was published in Trials, accessible at https://trialsjournal.biomedcentral.com/articles/10.1186/s13063-017-1894-7.

This update reports on the eligibility criteria expansion and on the adjunction of a cost-utility analysis. We widened the eligible population to patients with locally advanced or metastatic cancer including malignant hemopathies (except acute myeloid leukemia) and to patients in the first and second lines of oncologic treatment. We restricted the inclusion to patients with a Mini Mental State Examination score strictly over 24. In addition to the secondary outcomes outlined in the protocol, a medico-economic analysis has been added to evaluate both the health benefits and costs of the two strategies and calculate the incremental cost-utility ratio of the innovative program assessed, compared to the standard practice.

**Trial registration:**

ClinicalTrials.gov, NCT02829762. Registered on 29 June 2016.

## Background

PREDOMOS (PREcarious elderly patient supported for cancer: impact on quality of life of a DOMotic and remote assistance approach for Socially isolated elderly patients supported for locally advanced or metastatic cancer) is a randomized controlled trial aiming to evaluate the impact of establishing a Program of Social intervention associated with techniques of Domotic and Remote assistance (PS-DR) on the improvement of quality of life (QoL) at 3 months (primary endpoint) of older isolated patients, treated for locally advanced or metastatic cancer. This study will also assess the effect of the PS-DR on treatment compliance, autonomy maintenance, nutrition, mobility, toxicity, time to treatment failure, survival, social isolation, unexpected hospitalizations, and QoL at 6 months (secondary endpoints). In addition, PREDOMOS aims to identify individual geriatric characteristics (such as functional, nutritional, cognitive, and mobility status) that would be predictive of clinical outcomes.

As a reminder, 320 individuals are required to obtain 90% power to detect a 10-point difference (standard deviation 25) in QoL score between the two groups (20% loss to follow-up patients expected). Participants are randomized in a 1:1 allocation ratio, either in the control group, receiving usual care, or in the experimental group, receiving usual care associated to PS-DR.

The PREDOMOS study, supported by a grant from the French Ministry of Health, is coordinated by the Assistance Publique des Hôpitaux de Marseille (AP-HM, France) and includes multiple partners in oncologic and geriatric units across France, such as the South Alpes Intercommunal Hospital Center in Gap, the Saint Joseph Hospital and the Paoli Calmettes Institute in Marseille, the Nice University Hospital, the Saint Brieuc, Toulon, and Aix-en-Provence Hospital Centers, and the Saint Catherine Institute in Avignon.

The original study protocol was published by Crétel-Durand et al. [1]. Following the publication of the study protocol, amendments were made to reflect the changes to initial screening and patient recruitment and to add a cost-utility analysis, funded by the Fondation de France. These two changes are outlined below. The amendments of the protocol were approved by the Sud Mediterranée 1 Ethics Committee in June 2018.

## Methods/design

### Changes in eligibility criteria

To improve patient recruitment, which was difficult from the beginning of the study, the steering committee decided to modify the inclusion criteria. Tumor type restrictions were lifted, allowing patients with locally advanced or metastatic cancer from all organs, including malignant hemopathies, to enter the study. Only patients with acute leukemia will be excluded, in the hope of including patients with a lifespan of more than 6 months. Furthermore, the treatment conditions selected were expanded to include patients cared for in a second line of oncologic treatment. Due to the inclusion of patients with various cancers, we also specified allowed oncological treatments: new generation hormonotherapy, targeted therapy, or immunotherapy with or without, concomitant or not, radiotherapy.

Otherwise, to simplify the exclusion criteria, the steering committee added a new inclusion criterion to the protocol, “Mini Mental State Examination (MMSE) score strictly over 24,” to automatically exclude patients unable to complete the QoL questionnaire without help and patients with dementia. We also decided to delete the exclusion criterion “patient with psychiatric troubles” because in the absence of cognitive impairment these patients are potential candidates like the others.

The inclusion criteria now include the following: (1) patient aged 70 years or older; (2) socially isolated or at risk of social isolation (modified Medical Outcomes Study Social Support Survey (m-MOS-SS) under 80% and/or isolated patient (caregiver living more than 50 km from the patient) and/or primary caregiver to his/her spouse (for a spouse who has a limited autonomy, neurodegenerative disease, cancer, etc., implying regular medical care for at least 3 months); (3) Scale for detection of geriatric frailties in cancer patients over 70 years old (G8 ONCODAGE) score ≤ 14; (4) Eastern Cooperative Oncology Group Performance Status (ECOG-PS) ≤ 2; (5) Activities of daily living (ADLs) score ≥ 4; (6) MMSE > 24; (7) lifespan > 6 months; (8) with locally advanced or metastatic cancer, including malignant hemopathies (except acute leukemia); (9) in first or second line of oncologic treatment by chemotherapy, new generation hormonotherapy, targeted therapy, or immunotherapy with or without, concomitant or not, radiotherapy; (10) signed informed consent; and (11) affiliation to the French social security system.

The exclusion criteria include: (1) patients with a second cancer; (2) protected adults under guardianship or curatorship; and (3) patient to be immediately directed into a rehabilitation and recuperative care service to benefit from treatment.

### Secondary endpoints: adjunction of a cost-utility analysis (CUA)

Home medical care for older cancer patients can be a relevant response to the issue of increasing expenditures related to aging. Indeed, studies have shown that these approaches could be cost-effective; namely, they were effective on a set of clinical criteria at an acceptable cost to the community or the health care system [2–5]. For example, following an initial cost of implementation and equipment, home care interventions are accompanied by avoided costs of medical care (e.g., non-programmed re-hospitalizations, visits, examinations), as well as costs related to the financial burden for the patient. Consequently, cost analyses and cost-effectiveness analyses are crucial for planning proper distribution of health care resources and social/medical interventions in elderly cancer patient populations.

The aim of the economic analysis in the present study is to evaluate both the health benefits and costs of the two strategies and to calculate the incremental cost-effectiveness ratio of the innovative program assessed compared to the standard practice. As recommended by the French Health institution (HAS) [6], the clinical endpoint will be the number of quality-adjusted life years (QALYs) saved. These QALYs are determined by multiplying life years gained by a value of the utility associated (corresponding to patient’s quality of life) during the period under consideration [7]. As the scores in Quality of Life Questionnaire-Cancer (QLQ)-C30 are not utility-based, these scores will be mapped to the EuroQoL Five-levels (EQ-5D) [8, 9]. The QLQ-C30 at T3 will be used to cover the first 3 months (T0 to T3) and the QLQ-C30 at T6 to cover the period between T3 and T6. The time horizon will cover the period during which patients will be exposed to the intervention (i.e., 6 months). This will allow researchers to observe and collect prospectively all health and economic outcomes during the exposed period between the two groups. Therefore, no discounting will be necessary.

The perspective will be that of the society, and the following type of resources will be included in the cost analysis:

- Techniques of domotic and remote assistance (including the cost of equipment installation, renting, technical support, and uninstallation)
- Monthly telephone follow-up by the social worker
- Non-programmed hospitalizations in both groups (including emergency department visits)
- Prescribed exams, laboratory tests, and non-programmed medicines
- Unscheduled visits to the oncologist in both groups (including travel allowances for the patient)
- Costs incurred by the patients (in relation to out-of-pocket charges, extra travel costs). As patients are isolated, we hypothesized that caregiver costs would be negligible.

All resources will be observed, collected though the case report forms (CRFs), and valorized over the period between baseline and the 6 months follow-up. Several sources of unit costs will be investigated for this economic study based on HAS recommendations. Production costs through diagnostic-related groups from the Technical Agency for hospital information (ATIH) will be used for inpatient costs (sources: National Health system and hospital databases). For ambulatory care and examinations, national tariffs are recommended (sources: National databases for medical and paramedical acts, National official list prices for drugs and registers of pharmaceutical specialties, National Table for Biology).

Differences in health and costs outcomes’ means will be tested by using the Student *t* test (if normally distributed) and differences between distributions tested with the nonparametric Mann-Whitney *U* test (if means are not normally distributed). Data will be analyzed according to the intention-to-treat principle. Statistical methods will be investigated for dealing with missing data, and their potential impact (according to assumptions made to cope with missing data) on the CUA findings will be addressed in the sensitivity analysis. To address variability and to assess the generalizability of our results, univariate sensitivity analyses will be performed by varying health and cost variables one by one (including but not limited to incidence of sepsis, recurrence rates, or unit costs). The statistical uncertainty surrounding the incremental cost-utility ratio (due to sampling fluctuations for example) will be captured by a multivariate sensitivity analysis, using, among others, simulation methods such as non-parametric bootstrap methods [10]. In addition, cost-effectiveness acceptability curves will be constructed to represent decision uncertainty surrounding cost-effectiveness estimates [11].

## Conclusion

The changes to the PREDOMOS study do not influence the statistical analysis plan; the addition of a CUA results from additional funding, and the new patient inclusion/exclusion criteria will facilitate ongoing recruitment. The results of this study are expected to confirm that a PS-DR may be an interesting care management strategy for isolated older patients with cancer.

### Trial status

At the time of manuscript submission, the status of the trial is “recruiting.”

## Abbreviations

ADL: Activity of daily living
ANSM: French drug and device regulation agency (Agence Nationale de Sécurité du Médicament)
ATIH: Technical Agency for hospital information (Agence technique de l’information sur l’hospitalisation)
CEA: Cost-effectiveness analysis
CPPSM1: French ethics committee (Comité de Protection des Personnes Sud Méditerranée I)
ECOG-PS: Eastern Cooperative Oncology Group Performance Status
EQ-5D: EuroQol Five-levels
G8 ONCODAGE: Scale for detection of geriatric frailties in cancer patients over 70 years old
HAS: French Health institution (Haute Autorité de Santé)
m-MOS-SS: Modified Medical Outcomes Study Social Support Survey
MMSE: Mini Mental State Examination
PS-DR: Program of Social intervention associated with Domotic and Remote assistance techniques
QALY: Quality-adjusted life year
QoL: Quality of life
